# Objective Analysis of Age-Related Changes in the Superficial Musculoaponeurotic System in Japanese Females Using Computed Tomography

**DOI:** 10.1093/asjof/ojad043

**Published:** 2023-05-18

**Authors:** Itsuko Okuda, Katsuhiro Abe, Naoki Yoshioka, Takayoshi Komemushi, Masahiro Jinzaki, Hiroyuki Ohjimi

## Abstract

**Background:**

The superficial musculoaponeurotic system (SMAS) is an anatomical structure involved in facial aging.

**Objectives:**

This study focused on SMAS thickness and aimed to establish age-related changes in SMAS thickness.

**Methods:**

A total of 100 adult Japanese female participants (aged 20-79 years) were enrolled in the study. The participants were divided into 3 age groups: Y, M, and E, comprising 20 to 39, 40 to 59, and 60 to 79 years, respectively. Anatomical structures were used as landmarks to standardize the SMAS analysis sites. The SMAS in a fixed analysis area (FAA) was quantified using multi-detector computed tomography (MDCT), and the relationship between SMAS thickness and age, as well as BMI, was analyzed.

**Results:**

In 96 participants (four were excluded due to imaging artifacts), a moderate yet significant negative correlation was found between the average (A)-SMAS thickness within the FAA and age. The A-SMAS thickness in groups M and E was significantly lower than that of group Y, and the mean value of group E was significantly lower than that of group M. SMAS thickness was greater in the young population. The SMAS gradually became thinner with aging. A statistically significant correlation was not found between SMAS thickness and BMI.

**Conclusions:**

Using MDCT technology, age-related changes in SMAS were successfully analyzed. This highly objective analysis method corroborated the aesthetic surgical knowledge of the SMAS features related to facial aging. In clinical applications, our findings may help elucidate the mechanisms involved in facial aging.

**Level of Evidence: 3:**

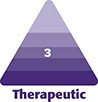

The superficial musculoaponeurotic system (SMAS) is a subcutaneous fibromuscular structure under the skin of the face that was first reported by Mitz and Peyronie in 1976.^1^ The SMAS keeps the cheeks in their normal position and allows the mimetic muscles to act in a coordinated manner with each other.^2^ The SMAS is a defined anatomical structure extending from the galea aponeurotica to the platysma muscle. Medially, it connects with major and minor zygomatic muscles and laterally to the parotid fascia.^3^ The supportive capacity of the SMAS is reduced over time due to soft-tissue damage caused by gravity, increased subcutaneous adipose tissue, musculofascial laxity, and solar elastosis.^4^ Furthermore, it is known that the SMAS gradually loosens with aging.^5^ In turn, this leads to the appearance of the signs of aging on the face.^2,4^

The SMAS is an important anatomical structure for understanding facelift procedures.^2,6,7^ Nevertheless, age-related changes in the SMAS itself have not yet been clarified. Therefore, we used computed tomography (CT) technology with a high spatial resolution to address this challenge.^3,5,8^ In anatomical imaging, the SMAS is recognizable as a linear subcutaneous fibromuscular structure.^3,5^ One limitation of SMAS analysis is that its thickness varies depending on the facial region.^9,10^ Therefore, it is necessary to standardize the analysis sites used to interrogate the SMAS, which are widely distributed across the face.

This study focused on SMAS thickness and aimed to establish age-related changes in SMAS thickness. SMAS thickness was analyzed, and the relationship between SMAS and age, as well as with BMI, was evaluated.

## METHODS

This retrospective study was approved by our institutional review board to use imaging data from previous multi-detector-row CT (MDCT), also known as spiral CT (No. 5-14-4). Participant anonymity was preserved, and the need for informed consent was waived owing to the retrospective nature of the study.

### CT Scan Technique

Scans of the whole face were performed with a tube voltage of 120 kVp, tube current of 200 mA, exposure time of 1.0 s, and slice thickness of 1 mm using a spiral CT with 320 detectors, namely a 320-MDCT scanner (Aquilion ONE; Toshiba Medical Systems, Tochigi, Japan). The field of view was set at 16.0 × 16.0 cm with 512 × 512 picture elements (pixels). One pixel was a square with 0.3125 mm (16.0 cm/512 pixels) sides.

All CT imaging data were transferred to a workstation (ZioCube; Ziosoft Inc., Tokyo, Japan), whereby 3-dimensional (3D) CT images were reconstructed. The workstation was equipped with several software applications to create reconstructed images and 3D CT images of the faces.

### Population Selection

The medical records of adult Japanese female patients who had undergone a previous medical examination at our institute were reviewed. Thus, this population comprised patients referred for head and neck CT for various indications. Participants with motion-related artifacts on CT images and a history of facial injuries and/or surgeries, including cosmetic procedures, were excluded from the study. Only participants without lesions that could influence the superficial facial structures and relevant bones (eg, negative studies and nasal sinusitis evaluations) were enrolled.

A total of 100 adult female participants aged 20 to 79 years (mean, 50.1 ± 18.0 years) comprised the study group. Participants were divided into 3 age groups: young (Y), middle-aged (M), and elderly (E), who were 20 to 39 (*n* = 34), 40 to 59 (*n* = 33), and 60 to 79 (*n* = 33) years old, respectively.

### CT Analysis of the Superficial Musculoaponeurotic System (SMAS)

This study was conducted involving a 2-step procedure. Different observers performed each step.

#### First Step: Attribution and Standardization of the Analysis Site of the SMAS

**Figure 1.**
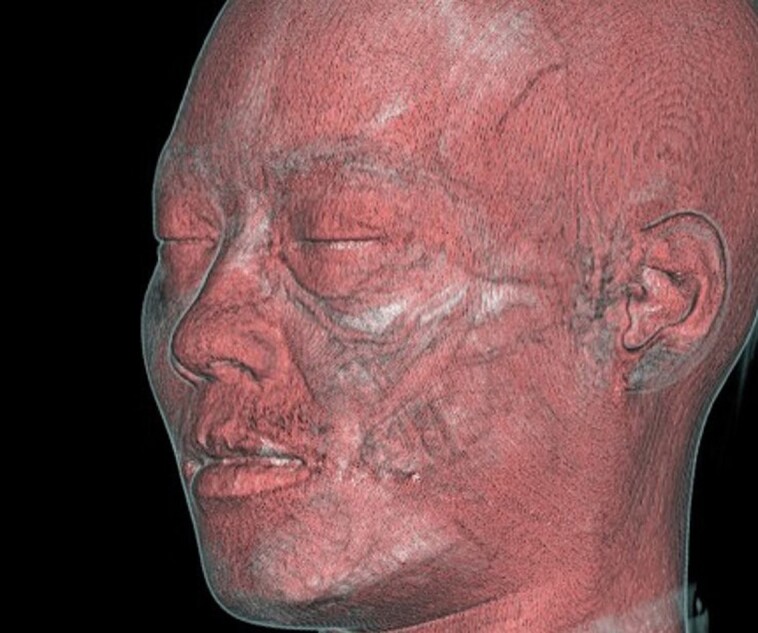
Three-dimensional computed tomography (3D CT) image. 3D CT was used to display the anatomical landmarks of the face, as well as the anatomical relationship of the subcutaneous structures. Created to target muscle density, it is superimposed with a transparent 3D CT image of the facial skin. Structures on the surface of the face and subcutaneous structures are simultaneously visible.

**Figure 2.**
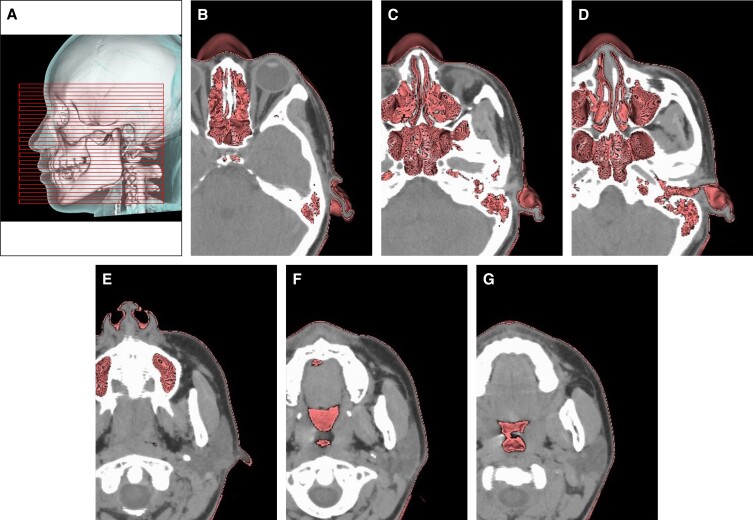
Creation of axial and coronal computed tomography (CT) images. (A) A leading three-dimensional CT(3D CT) image for accurately generating axial CT images. An infraorbitomeatal (IOM) line drawn between the infraorbital margin and external acoustic meatus is a global standard reference for CT. Axial CT images on lines parallel to the IOM line can be accurately created while simultaneously checking the existing anatomical structure. Coronal CT images were created in a plane orthogonal to axial CT images. (B-G) A guideline for creating accurate axial CT images. Axial CT images were cut from the whole-facial 3D CT image and a combination of several axial images of the left halves of the face. The accuracy of the axial CT images for each participant was confirmed using this reference image. The positional relationship between the structures of the facial surface and structures under the skin can be easily comprehended, and the superficial musculoaponeurotic system (SMAS) is visible as a linear structure in the subcutaneous adipose tissue. Its thickness varies depending on the facial region. (B) At the level of the central part of the eyeball: ethmoidal sinus is depicted. In the subcutaneous adipose tissue of the temporal region, membranous structures are visible. In order from the surface, the SMAS, superficial temporal fascia, and deep temporal fascia covering temporal muscle are distributed in layers. (C) At the level of the lower part of the eyeball: the maxillary and sphenoid sinuses are visible. In the subcutaneous adipose tissue of the temporal region, a membranous structure, the SMAS is recognized. (D) An image on IOM: the lower edge of the orbit, auricle and external acoustic meatus are visible. In the subcutaneous adipose tissue of the upper cheek, the SMAS is recognized. (E) At the level of the lower edge of the nose: the zygomatic major muscle and the anterior margin of the parotid gland are visible, and the SMAS is recognized in the subcutaneous adipose tissue between them. The masseter muscle exists deeply them. (F) At the level of the upper mandibula: the SMAS is recognized between the zygomatic major muscle and parotid gland. (G) At the level of the corner of the mouth: the SMAS is recognized between the major zygomatic muscle attaching to the orbicularis oris muscle and parotid gland.

The first step involved the attribution and standardization of the analysis sites of the SMAS. To determine the analysis site of the SMAS, 3D CT images that enabled an assessment of the anatomical relationship of the subcutaneous structures were created (Figure 1). Axial CT images were then created on lines parallel to the infraorbitomeatal (IOM) line drawn between the infraorbital margin and external acoustic meatus (Figure 2). From these images, the distribution of the SMAS and the anatomical relationship between the surrounding structures were obtained. The criteria for determining the analysis site of the SMAS were as follows: (1) the SMAS should be sandwiched between subcutaneous adipose tissues, and (2) there should be no contact between the SMAS and surrounding structures.

Anatomical landmarks were used to standardize the selected images and to improve the accuracy of the analysis. Coronal CT images were created in a plane orthogonal to axial CT images. The analysis and decision were performed by 2 observers (1 with 30 years of experience as a radiologist and another with 19 years of experience as a plastic surgeon and 8 years of experience as a radiologist) who consulted with each other.

#### Second Step: Quantitative Analysis of the SMAS

The second step was a quantitative analysis of the SMAS. The areas for quantitative analysis of the SMAS were set using the axial and coronal CT images selected in the first step. The subcutaneous adipose tissue containing the SMAS was isolated with a 9 × 3 mm rectangle, and this rectangular area was defined as the fixed analysis area (FAA). The area occupied by the SMAS within the FAA was quantitatively analyzed. The average SMAS (A-SMAS) thickness within the FAA was calculated from the area of the SMAS as follows:


$$A\text{-}\mathrm{SMAS}\;\mathrm{thickness}(\mathrm{mm})\;=\frac{\mathrm{Area}\;\mathrm{of}\;\mathrm{the}\;\mathrm{SMAS}}{9}$$

Quantitative analysis of the SMAS was performed using ImageJ software (National Institutes of Health, Bethesda, MD)^11^ with contrast enhancement and binarization. Two observers performed the analyses in consultation with each other: 1 with 23 years of experience as a radiological technician and 1 with 27 years of experience as a radiologist.

### BMI

The height (m) and body mass (kg) of participants enrolled in this study were reviewed from medical records, and BMI was calculated from them. The BMI was calculated as follows:


$$\mathrm{BMI}\;=\;\mathrm{kg}/m^{2}$$

### Statistical Analysis

The ages and BMIs of the participants are presented as mean ± standard deviation (SD) and range. Measured values of the A-SMAS thickness within the FAA are presented as mean (SD) values and range. Correlation analysis was performed to determine the relationship between A-SMAS thickness within the FAA and age, as well as A-SMAS thickness within the FAA and BMI. An independent-sample *t* test was used to compare the mean values of A-SMAS thickness among the 3 age groups. Moreover, inferences were made using these numerical values.

Statistical significance was set at *P* < .05. Statistical analyses were performed using StatMate V statistical software package (Nihon 3 B Scientific, Inc., Niigata, Japan).

## RESULTS

### CT Analysis of the SMAS

The CT images of 3 participants out of the total 100 female participants could not be analyzed due to metallic artifacts of caries treatment, and the CT images of 1 participant could not be analyzed due to misregistration artifacts caused by head movement during the CT scan. In the remaining 96 participants (aged 20 to 79 years; mean, 49.5 ± 18.0 years), the CT images were of high quality, and there was no deficiency of the SMAS. Analyses of the SMAS were feasible for the 96 participants aged 20 to 79 years (Table).

**Table.** Results of Quantitative Analysis of the SMAS in 96 Female Participants

Age groups	Participants (*n*)	Age (years)		BMI		A-SMAS thickness (mm)	
		Mean (SD)	Range	Mean (SD)	Range	Axial CT image	Coronal CT image
Y	34	28.8 ± 5.7	20-39	22.3 ± 4.5	15.8-30.8	1.16 ± 0.17	1.16 ± 0.18
M	32	51.7 ± 5.8	40-59	22.6 ± 4.7	17.3-32.9	1.00 ± 0.13	0.90 ± 0.12
E	30	70.6 ± 5.0	61-79	22.8 ± 4.4	18.4-31.5	0.87 ± 0.17	0.82 ± 0.19
Total	96	49.5 ± 18.0	20-79	22.5 ± 4.5	15.8-32.9	1.01 ± 0.19	0.97 ± 0.22

Values are *n*, mean (SD), or range. A-SMAS thickness, average of superficial musculoaponeurotic system (SMAS) thickness; CT, computed tomography; E, elderly (60-79 years old); M, middle-aged (40-59 years old); SD, standard deviation; Y, young (20-39 years old).

#### First Step: Attribution and Standardization of the Analysis Site of the SMAS

**Figure 3.**
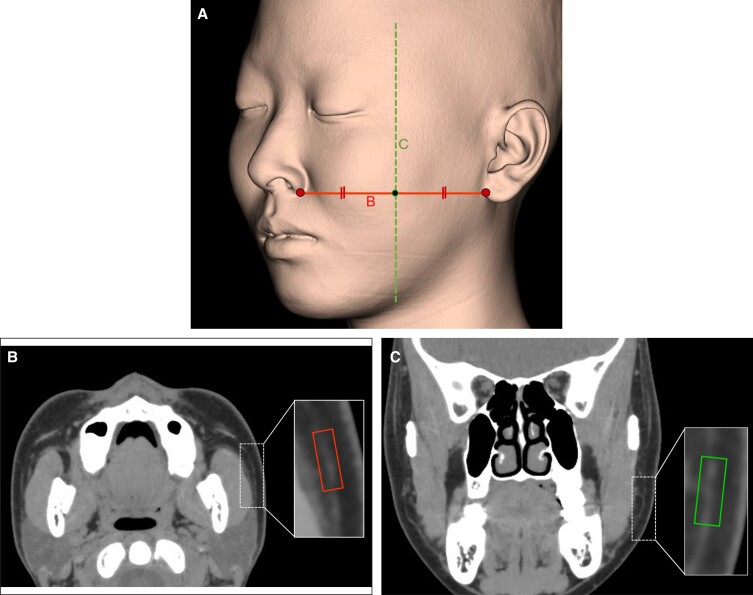
Quantitative analysis on axial and coronal computed tomography (CT) images of superficial musculoaponeurotic system (SMAS) analysis. (A) Three-dimensional (3D) CT image showing anatomical landmarks for standardization of the analysis site of the SMAS. The inferior margin of the nose and anterior margin of the earlobe were used as anatomical landmarks (large dots) for an axial CT image, and the center of the fixed analysis area (FAA; small dot) was installed at the midpoint connecting them. The horizontal line B indicates the location of the axial CT image. A coronal CT image was created at the location of the small dot in a plane orthogonal to the axial CT image along the vertical dotted line C. (B) Axial CT images for quantitative analysis of the SMAS: the subcutaneous adipose tissue containing the SMAS was delineated using a 9 × 3 mm rectangle, and this rectangular area was defined as the FAA. The subcutaneous adipose tissue of the cheek was sufficiently thick and determined to be optimal for SMAS analysis. Even within the FAA, SMAS thickness is not uniform. The area occupied by the SMAS within the FAA was quantitatively analyzed using ImageJ software with contrast enhancement and binarization. (C) Coronal CT images for quantitative analysis of the SMAS. Using the same method as for the axial CT image, the area occupied by the SMAS within the FAA was quantitatively analyzed using ImageJ software (National Institutes of Health).

An axial CT image at the level of the inferior border of the nose met the criteria for SMAS analysis. In all age groups, the subcutaneous adipose tissue of the cheek was sufficiently thick and determined to be optimal for SMAS analysis. To standardize the analysis site for SMAS, the inferior margin of the nose and the anterior margin of the earlobe were used as anatomical landmarks (Figure 3A). The FAA was installed at the midpoint between these points (Figure 3B). As shown in Figure 3C, the coronal CT image created in a plane orthogonal to the axial CT images met the criteria for SMAS analysis site selection. The midpoint of the FAA of the coronal CT image was installed at the same midpoint as that of the axial image.

#### Second Step: Quantitative Analysis of the SMAS

As shown in the Table, the mean (±SD) values of the A-SMAS thickness in the FAA on axial and coronal CT images of 96 participants were 1.01 ± 0.19 mm and 0.97 ± 0.22 mm, respectively.

**Figure 4.**
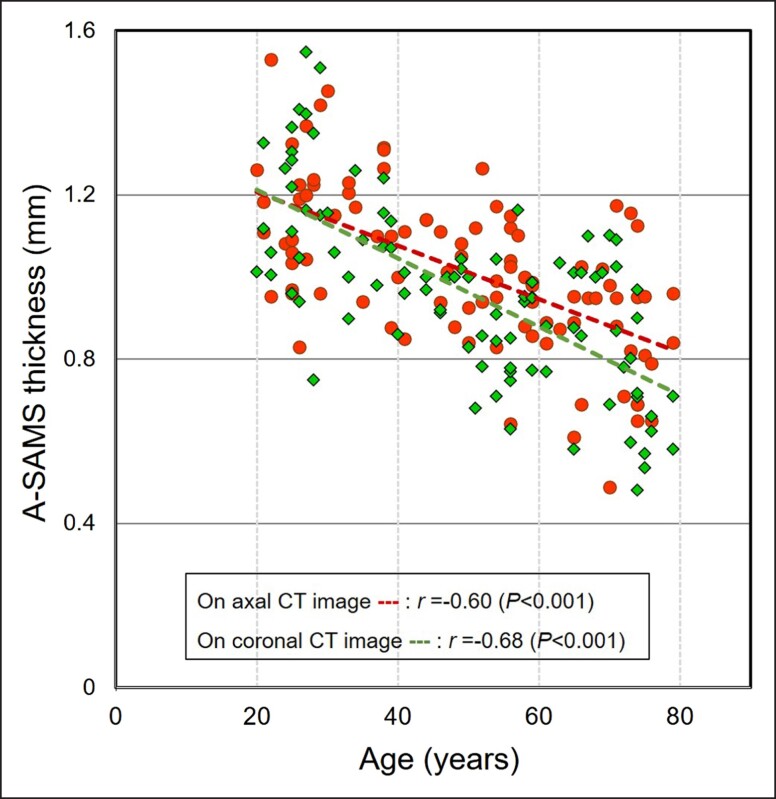
Correlation between average superficial musculoaponeurotic system (A-SMAS) thickness within fixed analysis area (FAA) and age. A moderate yet significant negative correlation was found between A-SMAS thickness (circle) in FAA on axial computed tomography (CT) images and age (*r* = −0.60, *P* < .001). A moderate yet significant negative correlation was found between A-SMAS thickness (rhombus) in FAA on coronal CT images and age (*r* = −0.68, *P* < .001). Specifically, the SMAS becomes thinner with aging.

**Analysis of the Relationship Between the A-SMAS Thickness Within the FAA and Age:** On axial and coronal CT images (Figure 4), our analysis found a moderate but significant negative correlation between A-SMAS thickness within the FAA and age (on axial CT images: *r* = −0.60, *P* < .001; on coronal CT images: *r* = −0.68, *P* < .001). With aging, the thickness of A-SMAS decreased.

**Figure 5.**
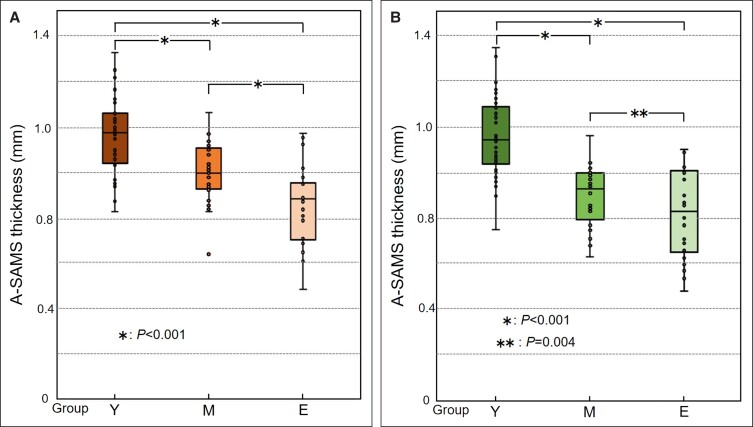
The average superficial musculoaponeurotic system (A-SMAS) thickness within the fixed analysis area (FAA) among the 3 age groups. (A) The A-SMAS thickness within the FAA on axial computed tomography (CT) images among the 3 age groups. The A-SMAS thickness in both groups M (*n* = 32; 1.00 ± 0.13 mm) and E (*n* = 30; 0.87 ± 0.17 mm) was significantly lower than the corresponding value in group Y (*n* = 34; 1.16 ± 0.17 mm; both *P* < .001). A-SMAS thickness was significantly lower in group E than the corresponding value in group M (*P* < .001). (B) The A-SMAS thickness within the FAA on coronal CT images among the 3 age groups. The A-SMAS thickness in both group M (*n* = 32; 0.90 ± 0.14 mm) and group E (*n* = 30; 0.82 ± 0.19 mm) was significantly lower than the corresponding value in group Y (*n* = 34; 1.16 ± 0.18 mm; both *P* < .001). A-SMAS thickness was significantly lower in group E than the corresponding value in group M (*P* = .004).

**The Difference in A-SMAS Thickness Among the 3 Age Groups:** As shown in Figure 5A, on axial CT images grouped according to the age of 96 participants, the mean (±SD) values of the A-SMAS thickness within the FAA of the 3 age groups were 1.16 ± 0.17 in group Y (*n* = 34), 1.00 ± 0.13 in group M (*n* = 32), and 0.87 ± 0.17 mm in group E (*n* = 30). As shown in Figure 5B, on coronal CT images, the mean A-SMAS thickness within the FAA was 1.16 ± 0.18 in group Y (*n* = 34), 0.90 ± 0.12 in group M (*n* = 32), and 0.82 ± 0.19 mm in group E (*n* = 30).

For both axial and coronal CT images, the A-SMAS thickness in groups M and E was significantly lower than that in group Y (*P* < .001). For both axial and coronal CT images, the mean value of group E was significantly lower than that of group M (*P* < .001 and *P* = .004, respectively). Specifically, it could be said that the SMAS was thicker in the younger population, and the SMAS gradually became thinner with age.

**Relationship Between A-SMAS thickness and BMI:** As shown in the Table, the mean (±SD) values of BMI of 96 participants were 22.5 ± 4.5. On axial and coronal CT images, a statistically significant correlation was not found between A-SMAS thickness and BMI (on axial CT images: *r* = −0.19, *P* = .15; on coronal CT images; *r* = −0.09, *P* = .48).

## DISCUSSION

Facial aging is caused by cumulative age-related changes in anatomical structures such as the skin, mimetic muscles, SMAS, retaining ligaments, and facial bones.^12,13^ We focused on SMAS thickness and analyzed its age-related changes. MDCT technology was used to analyze SMAS thickness accurately. Current CT technology provides high-resolution images, allowing detailed visualization of facial appearance and subcutaneous structure changes.^3,8^ The workstation used for data capture also housed an imaging analysis system that can create various 3D CT images.^3,5^

In this study, the field of view of CT images was set 16.0 × 16.0 cm with 512 × 512 pixels, resulting in a SMAS spatial resolution of 0.3125 mm (16.0 cm/512 pixels). Okuda et al performed anatomical radiological correlations of SMAS and proved that SMAS can be visualized with the spatial resolution from 0.35 to 0.39 mm.^3^ We used CT images with higher resolution, resulting in high-resolution CT images which were considered sufficient to analyze SMAS. The thickness of subcutaneous adipose tissue on the cheeks varied among individuals. Although the participant was slender, the thickness of the subcutaneous adipose tissue of the cheeks was more than 3 mm; thus, the short side of the FAA was set at 3 mm. The long sides of the FAA were considered integer multiples of 3 mm for the convenience of calculation. The subcutaneous adipose tissue and SMAS distributed beneath the skin were curved along the cheek contour, and the longest length as a rectangle with a 3 mm short side that could be taken in it was 9 mm.

The thickness of the SMAS varies according to the site of the face,^10,14^ and exhibits differences between individuals.^5^ It is said that the parotid-masseteric region is the thickest, the anterior cheek region is the thinnest, and the temporal region of the forehead is quite thick.^14^ Therefore, it was essential to standardize the analysis site of the SMAS. An IOM line drawn between the infraorbital margin and external acoustic meatus is a global standard for CT, and we accurately created axial CT images parallel to the IOM line. Axial images referenced the inferior margin of the nose, and the anterior margin of the earlobe was used for SMAS analysis. Using these anatomical structures as landmarks, the analysis site of the SMAS of the cheek could be standardized. We made it possible to quantify the thickness of the SMAS with high accuracy.

It was found that the SMAS gradually became thinner with aging. Specifically, the SMAS was thicker in the younger population but thinner in the elderly population. The SMAS comprises muscle cells, collagen fibers, and elastic fibers.^3,15^ These tissues are known to depreciate with aging.^8,16,17^ It is inferred that these tissue reductions lead to SMAS thinning. In addition, thinning of the SMAS with aging could lead to sagging cheeks due to loosening of its supportive force.^4,5^

In 2019, Okuda et al^5^ reported that SMAS laxity increases due to aging. Furthermore, in a report by Okuda et al^8^ in 2022, it was shown that the orbicularis oculi muscle became thinner due to aging. Additionally, it has been shown that the lateral part of the orbicularis oculi muscle, lateral canthal tendon, and major zygomatic muscle droop with aging when standing. These anatomical and functional changes may be involved in the mechanism of facial aging. Age-related thinning of the SMAS demonstrated in this study is consistent with these observations. It was suggested that thinning of the SMAS, including mimetic muscles, and drooping of the SMAS plane were involved in the characteristic signs of facial aging. On the other hand, a relationship between SMAS thinning and BMI was not confirmed in this study, which might be due to the narrow range of BMIs.

The facial nerve runs through the parotid gland in the lateral portion of the face and is relatively deep. After passing through the parotid gland, the facial nerve divides into 5 branches and runs in the superficial layer of the face, where these branches reach the mimetic muscles.^18,19^ Therefore, the vicinity of the zygomatic arch should be avoided in facelift procedures using the SMAS. Additionally, the SMAS should be excised from the parotid fascia in the parotid region.^19,20^ When excising thinned SMAS in elderly patients, it is necessary to pay attention to the parotid fistula due to potential damage to the parotid fascia.^6^ Furthermore, while performing facelift with the thinned SMAS, attention should be paid to the tension applied to the SMAS so as to not damage it.^21,22^ Due to concerns of SMAS becoming thinner with aging, it is necessary to confirm the allowable range of force and vector for SMASectomy based on patients’ age.

This study showed that the SMAS, like muscles of facial expression,^8^ become thinner with aging, suggesting that it is involved in mechanisms of facial aging. In the future, if the aging mechanism of SMAS is elucidated in more detail, it may be possible to apply it to nonsurgical facial rejuvenation therapy and help develop more effective and safer facelift surgeries.

This study has some limitations. First, a statistical analysis of the relationship between SMAS thickness and age was conducted using CT imaging data from a large number of participants in lieu of analyzing age-related changes over time in individual participants, as the study did not cover a sufficiently long period. Second, this study focused on Japanese female participants, and did not compare the findings to other racial groups. Third, we analyzed SMAS thickness in only 2 sections: axial and coronal CT images of the cheek. Nevertheless, we were able to recognize age-related changes in the SMAS. However, because the SMAS is widely distributed from the temporal region to the neck,^2,3^ further cross-sectional analysis would be desirable. Fourth, we focused on the relationship between age and SMAS thickness. However, various factors such as genetic makeup, race, aggregated sun exposure damage, and high BMI also contribute to the loosening of the SMAS.^4^ It is necessary to verify the relationship between these other factors and SMAS thickness in future studies. Fifth, we did not analyze the relationship between age-related changes in the SMAS and facial appearance. In the future, this relationship should be explored, and potential clinical applications investigated.

## CONCLUSIONS

We conducted a detailed analysis of age-related changes in the SMAS using MDCT technology. This evaluation method is highly objective, and the imaging analysis was corroborated by esthetic surgical knowledge about how SMAS features are related to facial aging. In clinical applications, our findings could contribute to the elucidation of the mechanisms involved in facial aging.
